# A Novel Minimal Invasive Mouse Model of Extracorporeal Circulation

**DOI:** 10.1155/2015/412319

**Published:** 2015-01-29

**Authors:** Shuhua Luo, Menglin Tang, Lei Du, Lina Gong, Jin Xu, Youwen Chen, Yabo Wang, Ke Lin, Qi An

**Affiliations:** ^1^Department of Cardiovascular Surgery, West China Hospital, Sichuan University, Chengdu, Sichuan 610041, China; ^2^Intensive Care Unit, West China Hospital, Sichuan University, Chengdu, Sichuan 610041, China; ^3^Department of Anesthesiology and Translational Neuroscience Center, West China Hospital, Sichuan University, Chengdu, Sichuan 610041, China; ^4^Department of Experimental Medicine, West China Hospital, Sichuan University, Chengdu, Sichuan 610041, China

## Abstract

Extracorporeal circulation (ECC) is necessary for conventional cardiac surgery and life support, but it often triggers systemic inflammation that can significantly damage tissue. Studies of ECC have been limited to large animals because of the complexity of the surgical procedures involved, which has hampered detailed understanding of ECC-induced injury. Here we describe a minimally invasive mouse model of ECC that may allow more extensive mechanistic studies. The right carotid artery and external jugular vein of anesthetized adult male C57BL/6 mice were cannulated to allow blood flow through a 1/32-inch external tube. All animals (*n* = 20) survived 30 min ECC and subsequent 60 min observation. Blood analysis after ECC showed significant increases in levels of tumor necrosis factor *α*, interleukin-6, and neutrophil elastase in plasma, lung, and renal tissues, as well as increases in plasma creatinine and cystatin C and decreases in the oxygenation index. Histopathology showed that ECC induced the expected lung inflammation, which included alveolar congestion, hemorrhage, neutrophil infiltration, and alveolar wall thickening; in renal tissue, ECC induced intracytoplasmic vacuolization, acute tubular necrosis, and epithelial swelling. Our results suggest that this novel, minimally invasive mouse model can recapitulate many of the clinical features of ECC-induced systemic inflammatory response and organ injury.

## 1. Introduction

Extracorporeal circulation (ECC) is essential for conventional cardiac surgery and life support. Despite the profound benefits that ECC has brought to patients around the world, it often triggers a vigorous systemic inflammatory response that can significantly reduce the quality of clinical outcomes [1]. This systemic inflammation is thought to be induced by contact between the blood and foreign surfaces of the ECC apparatus, which differ substantially from the normal vascular endothelium. During this inflammatory response, leukocytes, especially neutrophils, are activated and they adhere to vascular endothelial cells, subsequently migrating into the interstitium, where they may release proteases, free oxygen radicals, and proinflammatory factors that can damage tissues [2]. Improving clinical outcomes after ECC therefore depends on inhibiting this systemic inflammatory response [3].

Numerous studies in animal models have sought to clarify the mechanism of ECC-induced systemic inflammatory response in an effort to identify ways to inhibit it [4–9]. These models have been limited to larger animals, such as pigs, dogs, or lambs, because of the cost and infrastructure involved. While these studies have yielded important insights into the mechanism of ECC-induced inflammation, they are expensive, challenging to carry out with large numbers of animals, and limited by the lack of commercially available antibodies.

Our laboratory has been working to develop a much simpler, minimally invasive small-animal model that mimics the clinical effects of ECC. Recently we described a simplified rat model of arterial-venous partial bypass, and we showed that the bypass procedure mimics the strong systemic inflammatory response and acute lung injury observed in patients [10]. Here we apply the same approach to mice, for which transgenic technology is far more developed and a more extensive range of commercial antibodies are available. The result is a murine model of arterial-venous partial bypass that may allow detailed molecular studies of ECC-induced systemic inflammatory response. The model requires only minimally invasive microsurgical techniques and low priming volume, and it features a high survival rate.

## 2. Material and Methods

The study protocol was approved by the Animal Research Ethics Committee of Sichuan University.

### 2.1. Animals

Inbred male C57BL/6 mice aged 10–12 weeks and weighing 25–30 g were purchased from Sichuan University (Chengdu, China). All animals received standard care according to the US National Institutes of Health* Guide for the Care and Use of Laboratory Animals*.

### 2.2. Surgical Procedures and ECC

Animals were anesthetized by intraperitoneal administration of 0.2 mg/kg pentobarbital, intubated orotracheally with a 20-gauge angiocatheter (Becton Dickinson Medical Devices, USA), and then connected to a small rodent ventilator (Taimeng Keji, Chengdu, China). The respiratory rate was set at 130 breaths/min and the tidal volume was 0.5 mL of room air.

**Figure 1:**
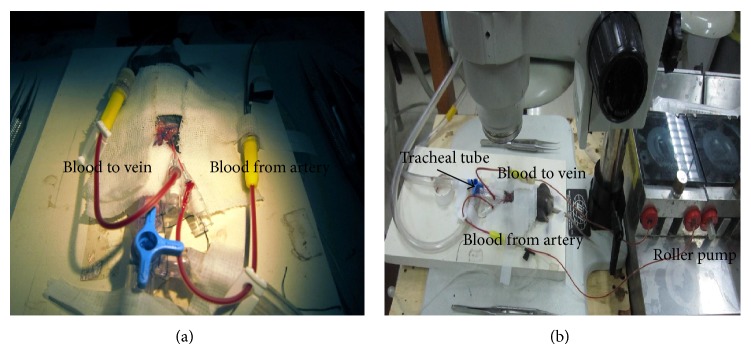
The mouse extracorporeal circulation circuit. Mice were anesthetized and mechanically ventilated. Both the right carotid artery (a.) and external jugular vein (v.) were cannulated and connected to a simplified ECC circuit comprising a sterile tube with a 1/32-inch inner diameter and a roller pump. Blood travelled from the right carotid (a. catheter) to the right external jugular (v. catheter).

Surgical procedures were performed with the aid of an operating microscope offering 10–40x magnification (Precision Stereo Zoom Trinocular Microscope III, World Precision Instruments, USA). After administration of heparin (500 U/kg), the right carotid artery was dissected out between the right strap muscle and sternocleidomastoid muscle. The omohyoid muscle was cut to improve visibility during the surgical procedures. One 24-gauge intravenous catheter (Becton Dickinson Medical Devices) was inserted into the right carotid artery and another into the external jugular vein, and then they were connected with a tube (inner diameter, 1/32 inch) primed with 0.4 mL normal saline and a roller pump (Stock II, Munich, Germany) in order to set up ECC (Figure 1).

During ECC, the flow rate was maintained at 5 mL/min, corresponding to approximately 25% of cardiac output [11]. ECC was performed for 30 min, after which the mice were observed for another 60 min.

### 2.3. Experimental Protocol

Mice were randomly divided into 3 groups (*n* = 20 in each). In the ECC group, mice were heparinized, cannulated, perfused for 30 min, and observed for 60 min, as described in Section 2.2. In the Sham group, mice were treated as in the ECC group, except that they were observed for 30 min instead of undergoing 30 min ECC. In the Naïve group, mice were anesthetized for 90 min, and samples were harvested immediately.

In both the ECC and Sham groups, mean arterial blood pressure (MAP) and heart rate were recorded before ECC (baseline), 15 min after the start of ECC, at the end of ECC, and 10 and 60 min after the end of ECC. Levels of circulating leukocytes and neutrophils were measured using an automated hematology analyzer (BC 3000 plus Mindray, Shenzhen, China) at 60 min after the end of ECC.

### 2.4. Levels of Proinflammatory Factors

To measure plasma markers of ECC-induced systemic inflammation in our model, blood samples were obtained at the end of the experiment, and plasma was separated by centrifuging the total sample for 15 min at 1000 ×g and 4°C. The plasma supernatant was removed and stored at −70°C until analysis. Levels of tumor necrosis factor-*α* (TNF-*α*), interleukin-6 (IL-6), and neutrophil elastase (NE) in plasma were assayed using the following commercial ELISA kits according to the manufacturers' instructions: TNF-*α* and IL-6, Thermo Scientific (Rockford, IL, USA); and NE, Cloud-Clone (Houston, TX, USA).

To measure ECC-induced organ damage in our model, the right lung and right kidney were harvested, homogenized, and centrifuged as described [12]. Briefly, tissues were harvested on ice immediately after euthanasia and weighed. An aliquot of tissue (50 mg) was cut into 1 mm^3^ pieces, added to 500 *μ*L normal saline, and homogenized. Samples were then left standing on ice for 5 min and centrifuged at 3500 ×g for 20 min at 40°C. Supernatants were transferred to Eppendorf tubes and stored at −70°C until analysis. Samples were assayed for TNF-*α*, IL-6, and NE as for plasma samples.

### 2.5. Assessment of Organ Function and Histological Injury

Arterial blood samples were taken from the carotid artery and O_2_ tension (PaO_2_) was measured both immediately at the end of ECC and 60 min after the end of ECC. The oxygen index was calculated from the equation OI = FiO_2_/PaO_2_, where FiO_2_ was assumed to be 0.21 [13]. To assess kidney function, serum levels of creatinine were measured using the Roche kinetic Jaffe method [14], and serum levels of cystatin C were measured using the latex particle-intensified immunity transmission turbidity method (Sichuan Maker Biotechnology Co., Ltd.) in the Clinical Biochemistry Laboratory of West China Hospital, Chengdu, China.

Organ damage was assessed histopathologically by harvesting the left lung and left kidney and fixing in 4% paraformaldehyde overnight at 4°C. Paraffin-embedded sections (3 *μ*m thick) were stained with hematoxylin and eosin and examined under a light microscope by a pathologist blinded to the experimental groups. Severity of lung injury was scored using a 5-point scale (0 = normal histology, 5 = most severe injury) that took into account alveolar congestion, hemorrhage, neutrophil accumulation in the airspace or vessel wall, alveolar wall thickness, and hyaline membrane formation [15]. Tubular injury was defined as epithelial swelling, loss of apical brush border, and intracytoplasmic vacuolization. Extent of acute tubular necrosis was assessed using a 4-point scale based on the percentage of affected tubules in four high-power fields of a light microscope: 0 points, no tubules affected; 1 point, <25% of tubules affected; 2 points, 25–50%; 3 points, 50–75%; and 4 points, >75% [16]. The mean score per section was calculated for each animal.

### 2.6. Statistical Analysis

Statistical analysis was performed using SPSS 11.01 (IBM, Chicago, IL, USA). Results were reported as mean ± standard error (SE). Intergroup comparisons for the same time point were assessed for significance using Student's *t*-test and a threshold of *P* < 0.05. Comparisons for different time points in the same group were assessed for significance using two-way repeated-measures ANOVA and a threshold of *P* < 0.05.

## 3. Results

### 3.1. Hemodynamics during ECC

All mice in the Sham and ECC groups survived the entire experimental procedure. MAP remained stable in both groups throughout the procedure and did not differ significantly (Table 1). This level was only approximately 10 mmHg lower than the value in the Naïve group, and the level in the ECC and Sham groups rapidly became similar to that in the Naïve group after ECC (*P* > 0.05).

Hematocrit (HCT) was significantly lower in the ECC group (0.31 ± 0.02) than in the Naïve group (0.40 ± 0.02, *P* < 0.05) and Sham group (0.37 ± 0.03, *P* < 0.05). Nevertheless, the value was still within clinically acceptable limits [17].

### 3.2. ECC-Induced Inflammatory Response and Organ Injury

**Figure 2:**
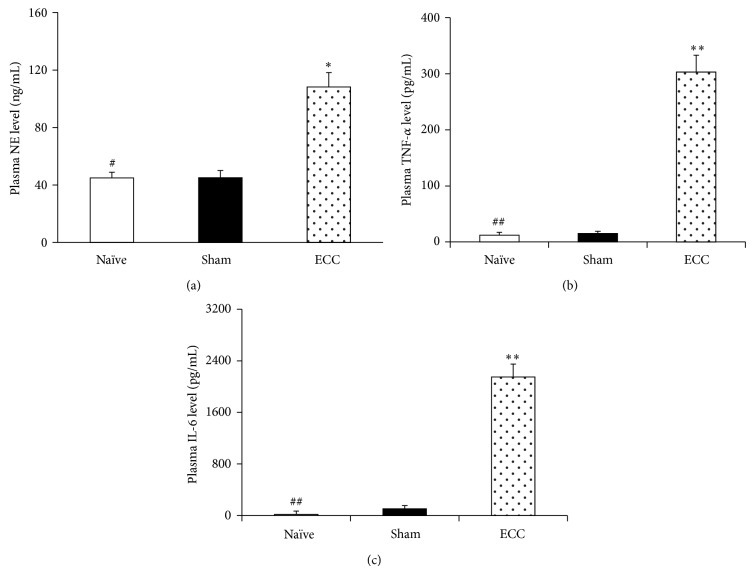
Systemic inflammatory response induced by ECC. Mice in the ECC group were perfused for 30 min in the ECC circuit shown in Figure 1 and then observed for 60 min. Mice in the Sham group were observed without ECC for 90 min. Mice in the Naïve group were anesthetized for 90 min, and samples were harvested at once. Systemic inflammatory response was assessed by measuring plasma levels of (a) NE, (b) IL-6, and (c) TNF-*α* at 60 min after the end of ECC. Each treatment group contained 20 animals. ECC, extracorporeal circulation; IL-6, interleukin-6; NE, neutrophil elastase; TNF-*α*, tumor necrosis factor-*α*. ^*^ *P* < 0.05 and ^**^ *P* < 0.01 versus Sham. ^#^ *P* < 0.05 and ^##^ *P* < 0.01 versus ECC.

**Figure 3:**
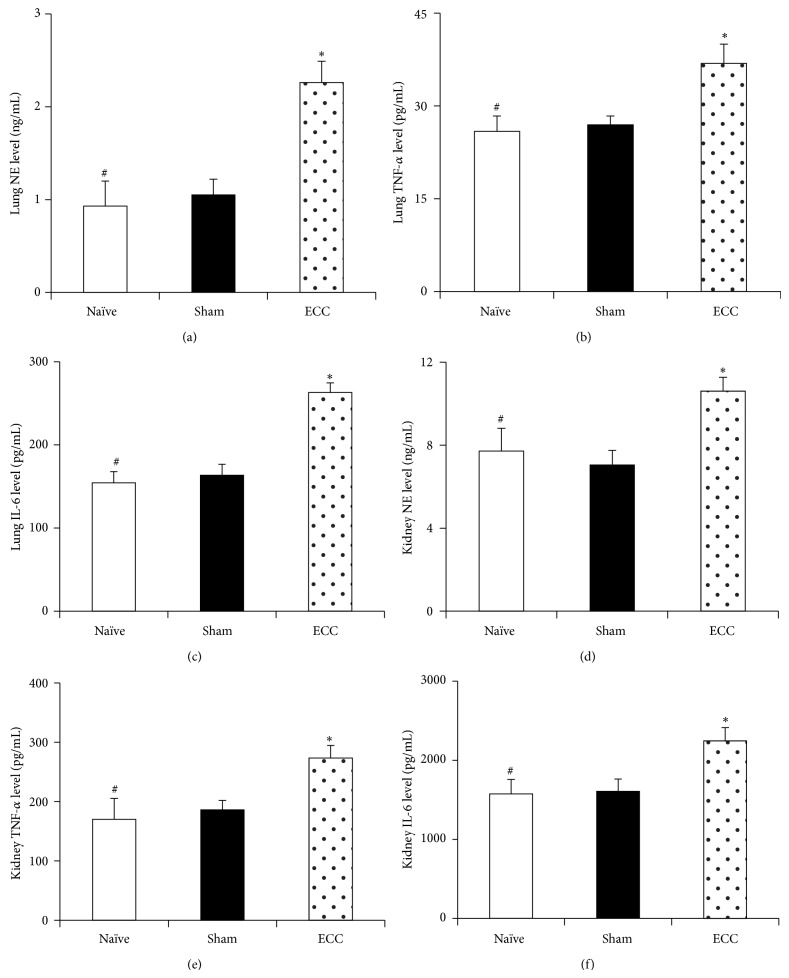
Inflammatory response in lung and kidney induced by ECC. Mice were treated as described in Figure 2, and lung and kidney tissues were harvested at the end of the experiment. Inflammatory response was assessed in lung (a–c) and kidney (d–f) by assaying levels of NE, TNF-*α*, and IL-6. ECC, extracorporeal circulation; IL-6, interleukin-6; NE, neutrophil elastase; TNF-*α*, tumor necrosis factor-*α*. ^*^ *P* < 0.05 and ^**^ *P* < 0.01 versus Sham. ^#^ *P* < 0.05 and ^##^ *P* < 0.01 versus ECC.

At 60 min after the end of ECC, the ECC group showed significantly higher levels of circulating leukocytes than the Sham group (13.2 versus 5.4 × 10^9^/L, *P* < 0.01) as well as higher levels of neutrophils (7.3 ± 0.6 versus 2.4 ± 0.3 × 10^9^/L, *P* < 0.01). Similarly, the ECC group showed significantly higher plasma levels of TNF-*α* (Figure 2(a)), IL-6 (Figure 2(b)), and NE (Figure 2(c)), as well as higher levels of all three proinflammatory markers in lung and kidney tissue (Figures 3(a)–3(c) and 3(d)–3(f)). Histopathological analysis was consistent with organ damage due to systemic inflammatory response.

**Figure 4:**
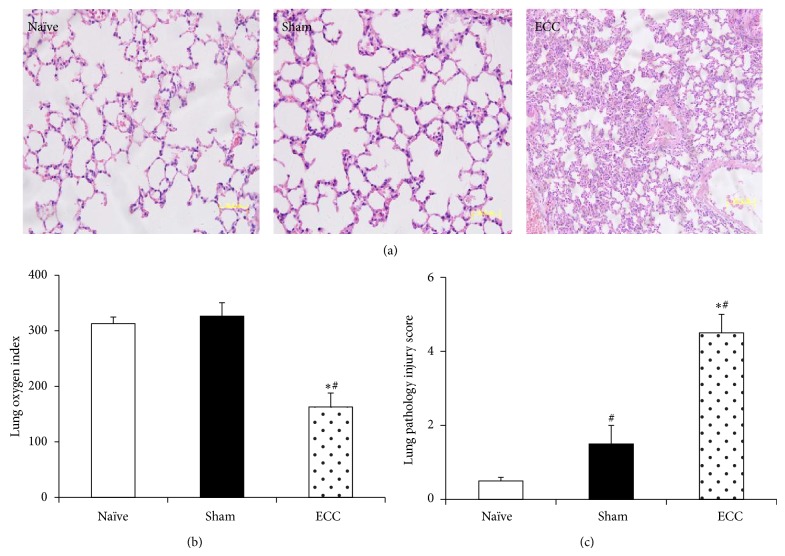
Lung function and tissue morphology after ECC. Mice were treated as described in Figure 2, and lung histopathology and arterial blood gas analysis were performed at 60 min after the end of ECC. (a) The upper lobe of the left lung was fixed in formaldehyde, sectioned, and stained with hematoxylin and eosin (*n* = 10; magnification, 40x). (b) The oxygenation index was calculated as FiO_2_/PaO_2_. (c) Acute lung injury was scored as described in Section 2. ^*^ *P* < 0.05 versus Sham, ^#^ *P* < 0.05 versus Naïve.

**Figure 5:**
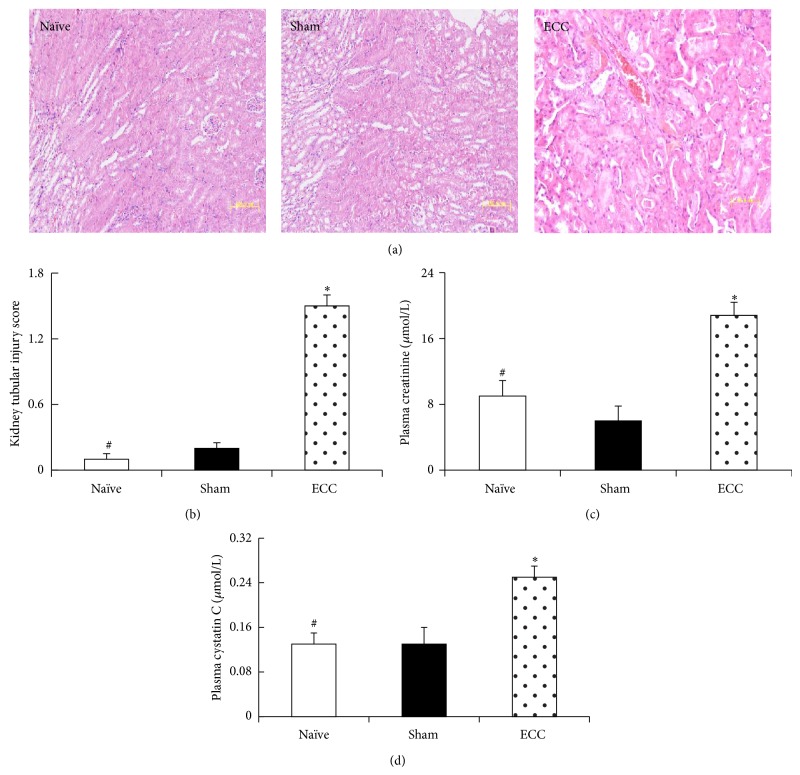
Kidney function and tissue morphology after ECC. Mice were treated as described in Figure 2, and kidney histopathology and blood analysis were performed at the end of the experiment. (a) Kidney sections were stained with hematoxylin and eosin and examined by a pathologist blinded to treatment groups. (b) Extent of renal tubular injury was scored as described in Section 2 (magnification, 40x). Blood was collected from the right carotid artery to allow serum assays of (c) creatinine and (d) cystatin C. ^*^ *P* < 0.05 versus Sham, ^#^ *P* < 0.05 versus ECC.

In contrast to the negligible tissue pathology observed in animals from the Sham and Naïve groups, mice in the ECC group showed pulmonary injury typical of ECC, characterized by edema, hemorrhage, alveolar wall thickening, and inflammatory cell infiltration into alveolar spaces (Figure 4(a)). Lung injury scores were significantly higher in the ECC group (4.50 ± 0.51) than in the Sham group (1.50 ± 0.12) or Naïve group (0.50 ± 0.03) (both *P* < 0.05; Figure 4(c)). Similarly, renal tubular damage scores were significantly higher in the ECC group (1.50 ± 0.12) than in the Sham group (0.21 ± 0.02, *P* < 0.05; Figure 5(d)). Tubular epithelial swelling was observed only in the ECC group (Figure 5(a)).

### 3.3. Effect of ECC on Organ Function

To complement histopathological assessment of ECC-induced organ injury, we assessed lung and kidney function by calculating the oxygenation index. Immediately after ECC, this index was similar between the Sham group (362 ± 20 mmHg) and ECC group (326 ± 25 mmHg, *P* < 0.05, Figure 3), but after an additional 60 min of observation the index in the ECC group decreased to a level significantly lower than that in the Sham group (163 ± 59 versus 326 ± 50 mmHg, *P* < 0.05; Figure 4(b)).

ECC also impaired kidney function. At 60 min after the end of ECC, the ECC group showed a significantly higher serum level of creatinine than the Sham group (16.71 ± 2.75 versus 8 ± 1.57 *μ*mol/L, *P* < 0.05), as well as a higher serum level of cystatin C (0.21 ± 0.12 versus 0.13 ± 0.09 *μ*mol/L, *P* < 0.05).

## 4. Discussion

Here we show that despite the small body weight, blood volume, and vessel diameter of mice, they may provide a reasonable model for ECC-induced systemic inflammatory response and related organ damage. The model reproducibly mimics the increases in blood and tissue markers of inflammation and the acute lung and kidney injury observed in the clinic. In addition, the model shows a high survival rate. This model may allow the rich array of mouse transgenic tools, knockout lines, and commercial antibodies to be brought to bear on the question of how ECC induces tissue damage.

To our knowledge, the smallest animal model of ECC is the rat, but this system is less reliable because it involves relatively high mortality [18, 19] and because donor blood from 1-2 rats is needed to prime the circuit, which may aggravate ECC-related pathophysiology [20–24]. The present model in mice is associated with high survival: all 20 mice subjected to ECC survived the entire experimental procedure, as did the 20 mice subjected to the Sham procedure. In addition, preliminary data suggest that most mice extubated approximately 1 hour after the end of ECC survive for at least another 24 hour (data not shown). In our model, the priming volume was substantially reduced with the aid of a 1/32-inch tube, which allowed volumes as small as 0.4 mL, corresponding to approximately 20% of the total mouse blood volume. Under these conditions without donor blood, the animals experienced only mild hemodilution and showed stable hemodynamics throughout the experiment. Even after removing the oxygenator from the circuit, arterial partial pressure of oxygen did not fall, suggesting that this arterial-venous partial bypass model can function reliably.

The specific clinical context of ECC, and the equipment involved, can vary substantially: it may or may not involve an oxygenator; it may or may not involve low blood flow, such as the case in left ventricular assistance and dialysis; and it may involve circulation from artery to vein or from one vein to another [25–27]. Nevertheless, a major problem common to all types of ECC is that contact of the blood with the foreign surface of the ECC equipment can induce a systemic inflammatory response that can cause adverse outcomes and compromise patient prognosis [28–36]. Since this systemic inflammation involves an increase in levels of circulating leukocytes and plasma levels of proinflammatory cytokines [37–41], the present study focused on the effects of ECC on levels of leukocytes, especially neutrophils, and plasma levels of TNF-*α*, IL-6, and NE. All these levels were significantly higher at 60 min after ECC in the ECC group than in the Sham or Naïve groups, reproducing well what has been reported in the clinic and in neonatal piglets subjected to ECC [42–45]. These results suggest that this arterial-venous partial bypass model can mimic the inflammatory response induced by ECC.

Importantly, lung and kidney tissues from our ECC animals showed the organ inflammation typical for ECC-induced injury, characterized by neutrophil infiltration and increase of levels of proinflammatory factors and NE [46–48]. ECC-induced organ injury was confirmed by scoring lung injury and renal tubular damage based on histopathology. In addition, our ECC mice showed a reduced PaO_2_/FiO_2_ ratio and elevated serum levels of cystatin C. In the clinic, ECC has been shown to induce a significant decrease in PaO_2_/FiO_2_ ratio 49–53, and acute renal dysfunction occurs in 5–31% of patients who undergo cardiac surgery involving ECC [49]. Our studies suggest that our mouse model recapitulates the acute lung and kidney injury frequently caused by ECC.

Despite its potential usefulness, our mouse model of ECC presents at least two important limitations that must be kept in mind. One is that it neglects other potential causes of systemic inflammatory response, such as ischemia-reperfusion injury. Another is that ligation of the right common carotid artery after ECC may induce gross neurological or histological abnormalities, which would limit the long-term usefulness of the model.

Within these limitations, our data suggest that we have developed a reliable, minimally invasive mouse model for studying how ECC leads to a systemic inflammatory response and subsequent acute lung and kidney injury in patients.

## Figures and Tables

**Table 1:** Hemodynamics in mice subjected to extracorporeal circulation (ECC).

Parameter	Group	Baseline	During ECC		After ECC	
			15 min	30 min	10 min	60 min
Mean arterial pressure, mmHg	ECC	63 ± 4	54 ± 6^a^	55 ± 7^a^	62 ± 10	67 ± 5
	Sham	64 ± 6	61 ± 8^b^	66 ± 9^b^	64 ± 4	68 ± 7

Heart rate, bpm	ECC	410 ± 10	420 ± 20	411 ± 10	413 ± 18	425 ± 22
	Sham	416 ± 15	412 ± 15	415 ± 12	415 ± 9	432 ± 11

Values reported as mean ± SE.

^a^ *P* < 0.05 versus Sham.

^b^ *P* < 0.05 versus ECC.
